# Trends and Disparities in Parkinson’s Disease Mortality in the United States with Predictions Using Machine Learning

**DOI:** 10.3390/neurosci6010006

**Published:** 2025-01-15

**Authors:** Henry Weresh, Kallin Hermann, Ali Al-Salahat, Amna Noor, Taylor Billion, Yu-Ting Chen, Abubakar Tauseef, Ali Bin Abdul Jabbar

**Affiliations:** 1 School of Medicine, Creighton University, Omaha, NE 68178, USA; henryweresh@creighton.edu (H.W.); kallinhermann@creighton.edu (K.H.); taylorbillion@creighton.edu (T.B.); 2 Neurology Department, Creighton University, Omaha, NE 68178, USA; yu-tingchen@creighton.edu; 3 Services Hospital, Lahore 40050, Pakistan; amnanoor101@gmail.com; 4 Department of Medicine, Creighton University, Omaha, NE 68178, USA; abubakartauseef@creighton.edu (A.T.); alibinabduljabbar@creighton.edu (A.B.A.J.)

**Keywords:** Parkinson disease, mortality, healthcare disparities, Parkinson disease mortality, machine learning

## Abstract

Background: Parkinson’s disease (PD) is a progressive neurodegenerative condition characterized by the degradation of dopaminergic pathways in the brain. As the population in the United States continues to age, it is essential to understand the trends in mortality related to PD. This analysis of PD’s mortality characterizes temporal shifts, examines demographic and regional differences, and provides machine-learning predictions. Methods: PD-related deaths in the United States were gathered from CDC WONDER. Age-adjusted mortality rates (AAMR) were collected, and trends were analyzed based on gender, race, region, age, and place of death. Annual percent change and average annual percent change were calculated using Joinpoint Regression program. Forecasts were obtained using the optimal Autoregressive Integrated Moving Average (ARIMA) model. Results: Overall mortality rate due to Parkinson’s increased from 1999 to 2022. Male gender, White race, Southern region, and older ages were associated with higher mortality compared to other groups. Deaths at home decreased and hospice deaths increased during the study period. Conclusions: This study highlights the increasing rate of PD AAMR and how it may become even more prevalent with time, emphasizing the value of increasing knowledge surrounding the disease and its trends to better prepare health systems and individual families for the burden of PD.

## 1. Introduction

Parkinson’s disease (PD) is a progressive neurodegenerative condition characterized by degradation of dopaminergic pathways in the substantia nigra pars compacta [1,2]. While PD is often described as idiopathic, evidence suggests that genetic and environmental factors contribute to its development [3]. The key features of PD include motor symptoms that are characterized by bradykinesia, resting tremor, postural instability, and muscular rigidity, along with non-motor symptoms like autonomic dysfunction, depression, anxiety, and sleep disorders [4]. The slow onset and progressive nature of PD has intense impacts on the quality of life for those experiencing the disease [5]. 

It is important to understand the trends in PD mortality because it is the second most common neurodegenerative disorder, following Alzheimer’s disease [6]. Furthermore, the prevalence of PD has been increasing exponentially [7]. Roughly 0.3% of the general population has PD, with greater prevalence among men compared to women, and adults over the age of 60 [6]. Not only is PD one of the most common neurodegenerative diseases, but it is also one of the most difficult to diagnose and treat [8]. Tremors can take years to appear after the onset of neuron loss, and every patient requires individualized management [8]. Nevertheless, recent studies have suggested that imaging modalities such as positron emission tomography can assist in the early diagnosis and thus treatment of PD [9]. Dopamine replacement therapy has been a core treatment for PD since its clinical introduction in 1961 [10]. Therapy with levodopa has its challenges because while it provides symptomatic relief, it does not delay neurodegeneration. Patients can require more frequent and greater doses with longer use and may develop wearing-off effects or dyskinesia [11]. Deep brain stimulation is another potential treatment for PD that has been used since 1986 to decrease motor symptoms, but it has financial costs that can pose a significant barrier to treatment [12]. 

The various motor and non-motor symptoms associated with PD can shorten life expectancy and lead to complications. Some of the leading causes of death among PD patients include cardiovascular disease, pneumonia, and infection [13]. As the population in the United States continues to age, it is essential to understand the trends in mortality related to PD. However, the literature lacks data on the recent and current burden of PD on the US population. Using the CDC Wide-ranging Online Data for Epidemiologic Research (WONDER), a public resource offering access to a wide array of public health information, this study attempts to elucidate PD’s most recent trends. This analysis of PD’s mortality characterizes temporal shifts, examines demographic and regional differences, and investigates progress in combating PD mortality. Understanding trends in PD mortality in the United States provides healthcare providers and policymakers insight into improving the ways we care for individuals with PD. This is of utmost importance as the burden of PD on our healthcare system continues to grow.

## 2. Methods

### 2.1. Study Design and Database

Information regarding PD-related deaths in the United States was abstracted from CDC WONDER [14]. The Multiple Cause-of-Death Public Use Records were assessed for PD as a contributing cause of death nationwide. This database has been utilized in similar studies for mortality related to heart failure [15]. PD-related mortality was found using the 10th Revision of the International Classification of Diseases (ICD) code G20 in patients 65 years and older. We restricted the study to this age group as PD mortality most commonly occurs in elderly populations. Given that the information taken from the CDC WONDER database is anonymous and publicly available, this study was exempt from institutional review board approval.

### 2.2. Demographic and Geographical Study Groups

This study gathered data on PD-related deaths from 1999 to 2022. Demographic information, including age, gender, race, census region, place of death, and states, were collected. Age groups were defined as 65–74, 75–84, and 85+ years of age. Race was defined and grouped as non-Hispanic (NH) White, NH Black, NH American Indian or Alaska Native, and Hispanic or Latino. Census regions were divided into Northeast, Midwest, South, and West according to the Census Bureau definitions. Places of death included medical facilities (inpatient, outpatient, or ER, dead on arrival), decedent’s home, hospice facility, nursing home or long-term care, and others. There were no data available regarding deaths within a hospice facility from 1999 to 2002.

### 2.3. Statistical Analysis

Crude mortality rates were calculated by dividing the total number of PD-related deaths each year by the corresponding United States population. Age-adjusted mortality rates (AAMR) were standardized using the 2000 United States standard population as previously described [16]. The Joinpoint Regression Program (version 4.9.0.0) determined trends in PD-related mortality within the study period [17]. Joinpoint finds significant changes in annual mortality trends and generates linear models depicting these temporal differences. Annual percent change (APC) with 95% confidence intervals (CI) for AAMR was calculated from Joinpoint using the Monte Carlo permutation test. The weighted averages of the APCs were calculated and reported as AAPCs with a corresponding 95% CI serving as a summary of the mortality trend for the entire study duration. Using a two-tailed *t*-test, APCs and AAPCs were increasing or decreasing if the slope measuring the change in mortality was significantly different from zero. Statistical significance was set at *p* ≤ 0.05 (represented by an asterisk ‘*’ in results and figures).

For predictive time series analysis, the Autoregressive Integrated Moving Average (ARIMA) model was utilized to forecast mortality rates until 2030 using non-stationary data. The ARIMA model was chosen for its effectiveness in handling non-stationary time series data and its widespread application in healthcare forecasting [18,19]. This model offers a more detailed understanding of time-dependent patterns compared to other alternatives [18,19]. 

An optimal ARIMA model was determined using the auto ARIMA function based on the Bayesian Information Criterion (BIC) and was then fitted to the data. The residuals of the model were assessed for white noise using the Ljung–Box test [18]. Additionally, the robustness of the model was validated through time series cross-validation (n = 10), with the Root Mean Squared Error (RMSE) reported to indicate accuracy [20].

## 3. Results

### 3.1. Overall (Figure 1a Shows Trend of Overall Changes in AAMR)

In the United States from 1999 to 2022, there were a total of 947,272 deaths due to Parkinson’s Disease.

**Figure 1:**
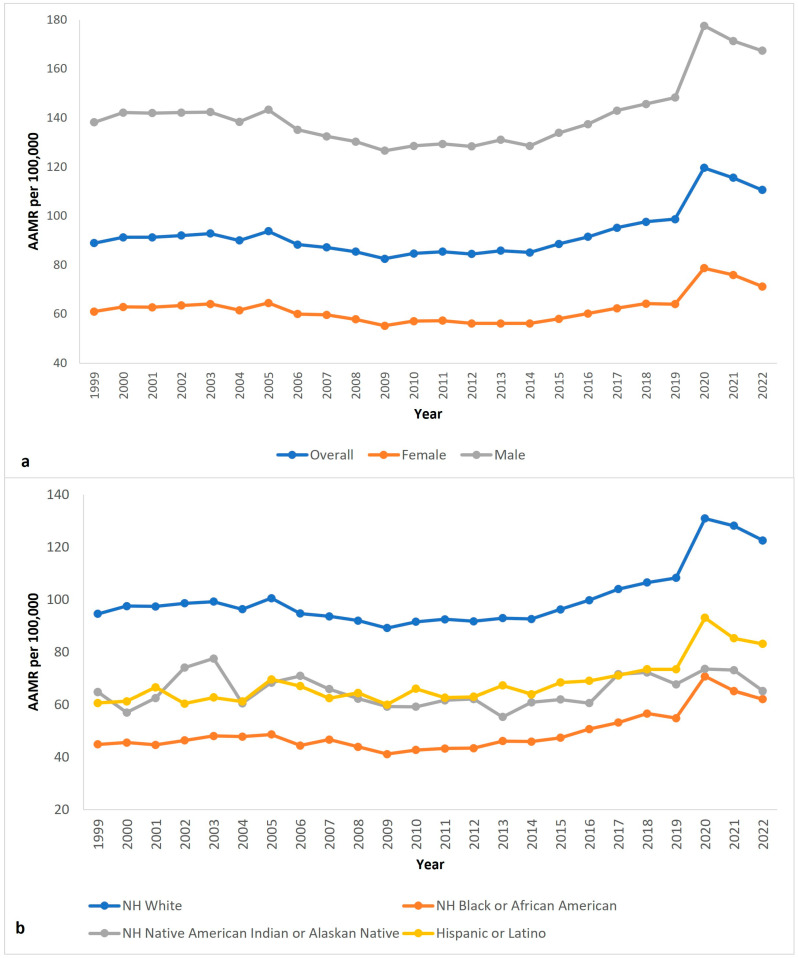
Parkinson’s disease age-adjusted mortality rate per 100,000 people in US from 1999 to 2022, (**a**) overall and stratified by biological sex; (**b**) stratified by race.

From 1999 to 2022, overall age-adjusted mortality rates (AAMR) increased, from 88.9 (95% CI 87.9 to 89.9) in 1999 to 110.6 (95% CI 109.7 to 111.5) in 2022 (AAPC 1.06 (95% CI 0.79 to 1.34)* (Supplementary Table S1, Figure 1a). The AAMR initially decreased from 1999 to 2014 (APC −0.61, 95% CI −1.17 to −0.17)*. The APC then increased to 4.26 (95% CI 3.30 to 5.55) * from 2014 to 2022 (Supplemental Figure S1). Overall, AAMR was lowest in 2009 at 98.7 and rose as high as 119.6 in 2020. 

### 3.2. Biological Sex (Figure 1a Shows Trend of Sex Stratified Changes)

Parkinson’s Disease was associated with 560,013 (59.1%) deaths in men and 387,259 (40.9%) in women in the United States from 1999 to 2022. The AAMR in men stayed higher throughout the study period, and this difference worsened during 2020–2022 (Supplementary Table S1).

From 1999 to 2022, the AAMR increased in men from 138.2 (95% CI 136.1 to 140.3) in 1999 to 167.4 (95% CI 165.6 to 169.1) in 2022 (Supplementary Table S1, Figure 1a). The AAPC of this period was 0.90 (95% CI 0.64 to 1.18). From 1999 to 2014, the APC in AAMR was −0.80 (95% CI −1.36 to −0.32)*. The APC then accelerated to 4.18 (95% CI 3.23 to 5.54)* from 2014 to 2022 (Supplementary Figure S1).

Women had lower AAMR compared to men. From 1999 to 2022, the AAMR in women increased from 61.0 (95% 60.0 to 62.1) in 1999 to 71.2 (95% CI 70.2 to 72.1) in 2022 (Supplementary Table S1, Supplemental Figure S1). The AAPC of this time period was 0.79 (95% CI 0.50 to 1.11)*. From 1999 to 2014, the APC in AAMR was −0.95 (95% CI −1.61 to −0.45)*. The APC then accelerated to 4.15 (95% CI 2.99 to 5.88)* from 2014 to 2022 (Supplementary Figure S1). 

### 3.3. Race (Figure 1b Shows Trend of Race Stratified Changes)

PD-associated mortality was found in 833,744 (88.0%) deaths among non-Hispanic (NH) White individuals, 41,082 (4.3%) deaths among NH Black or African American individuals, 2905 (0.3%) deaths among NH American Indian or Alaskan Native individuals, and 46,227 (4.9%) deaths among Hispanic or Latino individuals. Not only were the total deaths highest in the NH White population, but the AAMR was substantially higher in this group each year as well.

The NH White population had the highest AAMR, which increased from 94.5 (95% CI 93.4 to 95.6) in 1999 to 122.5 (95% CI 121.4 to 123.6) in 2022 and an AAPC of 1.23 (95% CI 0.97 to 1.51)* (Supplementary Table S2, Figure 1b). The APC was −0.48 (95% CI −1.02 to −0.04)* from 1999 to 2014, which increased to 4.52 (95% CI 3.56 to 5.86)* from 2014 to 2022 (Supplementary Figure S2). 

The NH Black or African American population had the lowest AAMR, which increased from 44.8 (95% CI 42.3 to 47.4) in 1999 to 62.1 (95% CI 59.8 to 64.4) in 2022 and an AAPC of 1.56 (95% CI 1.12 to 2.02)* (Supplementary Table S2, Figure 1b). The APC was −0.71 (95% CI −2.08 to 0.26) from 1999 to 2012, which increased to 4.58 (95% CI 3.47 to 6.25)* from 2014 to 2022 (Supplementary Figure S2). 

The NH American Indian or Alaskan Native population had an AAMR of 64.8 (95% CI 50.3 to 82.2) in 1999, which slightly increased to 65.2 (95% CI 55.9 to 74.4) in 2022 and an AAPC of 0.26 (95% CI −0.51 to 1.12) (Supplementary Table S2, Figure 1b). From 1999 to 2015, Non-Hispanic American Indian or Alaskan Native individuals had an APC of −0.86 (95% CI −7.51 to 0.72), which increased to 2.03 (95% 0.24 to 8.94)* from 2013 to 2022 (Supplementary Figure S2). 

The Hispanic or Latino population had an AAMR of 60.6 (95% CI 56.4 to 64.8) in 1999, which increased to 83.1 (95% CI 80.4 to 85.8) in 2022, with an AAPC of 1.30 (95% CI 0.87 to 1.85)* (Supplementary Table S2, Figure 1b). From 1999 to 2017, Hispanic or Latino people had an APC of 0.50 (95% CI −0.39 to 1.51) from 1999 to 2017, which increased to 9.46 (95% CI −0.49 to 11.98) from 2017 to 2020, and then decelerated to −3.15 (95% CI −8.24 to 4.58) from 2020 to 2022 (Supplementary Figure S2).

### 3.4. Region (Figure 2a Shows Trend of Region-Stratified Changes)

Throughout the duration of this study, all census regions saw an increase in Parkinson’s Disease AAMR. The South saw the greatest change, followed by the Midwest, West, and finally, the Northeast. Total deaths in the South were 328,529 (34.7%), Midwest were 234,451 (24.8), West were 211,555 (22.3%), and Northeast were 172,737 (18.2%).

**Figure 2:**
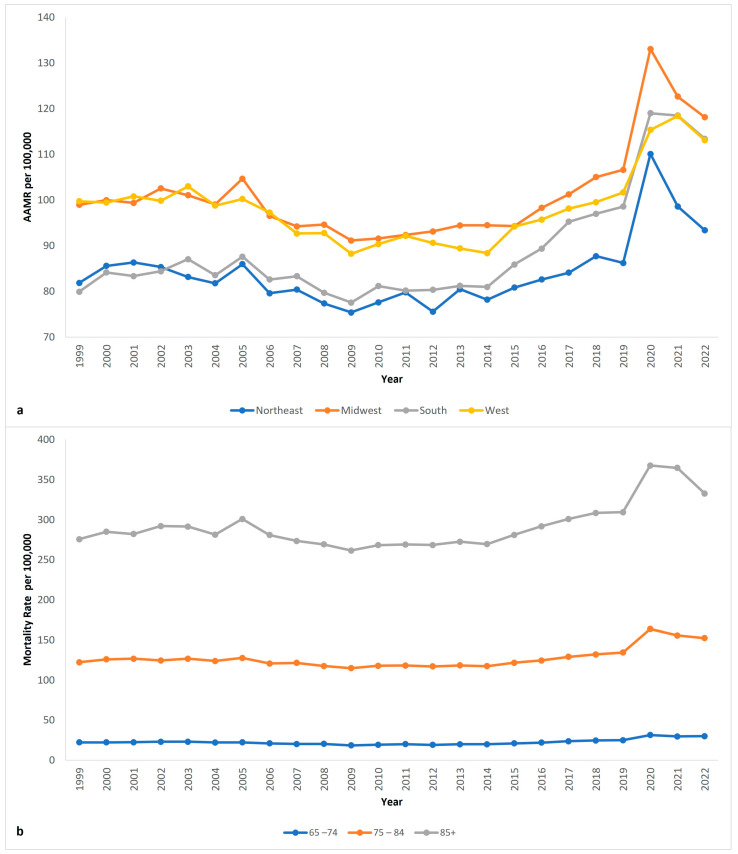
Parkinson’s disease age-adjusted mortality rate per 100,000 people in US from 1999 to 2022, (**a**) stratified by US census regions; (**b**) stratified by age groups.

AAMR in the South increased from 79.9 (95% CI 78.3 to 81.5) to 113.4 (95% CI 111.9 to 114.9) (Supplementary Table S3, Figure 2a). AAPC was 1.58 (95% CI 1.30 to 1.87)* from 1999 to 2022. APC was −0.28 (95% CI −0.88 to 0.22) from 1999 to 2014 before increasing to 5.16 (95% CI 4.16 to 6.54)* from 2014 to 2022 (Supplementary Figure S3). 

AAMR in the Midwest increased from 99.0 (95% CI 96.8 to 101.1) to 118.1 (95% CI 116.1 to 120.2) (Supplementary Table S4, Figure 2a). AAPC was 0.70 (95% CI 0.33 to 1.06)* from 1999 to 2022. APC was −0.52 (95% CI −1.02 to −0.06)* from 1999 to 2016, rose to 8.00 (95% CI 0.01 to 12.17)* from 2016 to 2020, and dropped to −2.93 (95% CI −7.58 to 4.08) from 2020 to 2022 (Supplementary Figure S3).

AAMR in the West increased from 99.7 (95% CI 97.3 to 102.1) to 113.1 (95% CI 111.2 to 115.0) (Table S4, Figure 2a). AAPC was 0.61 (95% CI 0.37 to 0.86)* from 1999 to 2022. APC was −0.97 (95% CI −1.51 to −0.53)* from 1999 to 2014 before increasing to 3.64 (95% CI 2.74 to 5.02)* from 2014 to 2022 (Supplementary Figure S3).

AAMR in the Northeast increased from 81.9 (95% CI 79.8 to 83.9) to 93.4 (95% CI 91.5 to 95.3) (Supplemental Table S3, Figure 2a). AAPC was 0.45 (95% CI 0.00 to 0.86) from 1999 to 2022. APC was −0.50 (95% CI −1.14 to 0.04) from 1999 to 2016, rose to 7.41 (95% CI −0.46 to 11.79) from 2016 to 2020, and dropped to −4.76 (95% CI −10.40 to 3.17) from 2020 to 2022 (Supplementary Figure S3).

### 3.5. Age Groups (Figure 2b Shows Trend of Age-Group-Stratified Changes)

From 1999 to 2022, the population of individuals 85 years and older had the highest overall crude mortality rate, which increased from 275.61 (95% CI 270.56 to 280.66) in 1999 to 332.60 (95% CI 328.16 to 337.04) in 2022 (mention AAPC in bracket). The crude mortality rate also increased in the other age groups. In the 65–74-year-old age group, the crude mortality rate was 22.26 (95% CI 21.58 to 22.94) in 1999, increasing to 30.09 (95% CI 29.51 to 30.68) in 2022. In the 75–84-year-old age group, the crude mortality rate was 122.25 (95% CI 120.29 to 124.21) in 1999, increasing to 152.36 (95% CI 150.54 to 154.19) in 2022 (Supplementary Table S4, Figure 2b).

### 3.6. State-Level (Figure 3 Shows Maps of State Level Changes)

The state-level AAMRs at the beginning of the study period varied from 134.5 (95% CI 108.5 to 160.5) in Vermont to 65.83 (95% CI 62.6 to 69.1) in New York. The top five states regarding PD-related mortality included Vermont, Minnesota, Nebraska, Alaska, and Washington. Conversely, the five states with the lowest AAMR included Arkansas, Louisiana, Florida, Alabama, and New York. The District of Columbia had the second lowest AAMR of 66.6 (95% CI 49.0 to 88.6) slightly above that of New York in 1999 (Supplementary Table S5).

Looking at 2020, the largest AAMR was 163.1 (95% CI 148.7 to 177.5) in Nebraska, while the smallest was 72.7 (95% CI 62.5 to 82.9) in Hawaii. The five states with the largest AAMRs included Nebraska, Minnesota, South Dakota, Oklahoma, and Iowa. On the other end, the states providing the lowest PD mortality rates were Florida, New York, Alaska, Nevada, and Hawaii. The District of Columbia had the fourth lowest AAMR of 93.2 (95% CI 73.9 to 116.0) in 2020 (Supplementary Table S5).

From 1999 to 2019, Alaska saw the largest decrease in AAMR from 113.1 (95% CI 74.5 to 164.5) to 70.0 (95% CI 51.3 to 93.4) (Supplementary Figures S4 and S5). In contrast, Tennessee had the largest increase in AAMR from 1999 to 2019, increasing from 77.3 (95% CI 70.7 to 83.9) to 118.2 (95% CI 111.5 to 124.9). 

From 2019 to 2020, Hawaii had the greatest decline in AAMR, dropping from 85.0 (95% CI 73.7 to 96.3) to 72.7 (95% CI 62.5 to 82.9) (Supplementary Figures S4 and S5). Conversely, South Dakota saw the greatest rise in AAMR, jumping from 113.6 (95% CI 96.0 to 131.2) to 155.5 (95% CI 135.0 to 176).

### 3.7. Place of Death

The number of Parkinson’s deaths with a known location was 945,265 (99.79% of the total). A total of 709,913 (74.94%) deaths occurred outside medical facilities (42.70% nursing home/long-term care, 27.06% home, 5.19% hospice), and 182,615 (19.28%) deaths occurred within medical facilities (16.73% inpatient, 2.33% outpatient/ER, 2.18% dead on arrival). Lastly, 52,737 (5.57%) deaths occurred in unknown/other places. From 1999 to 2022, deaths at home increased by 434.06%, and from 2003 to 2022, deaths in hospice increased by 6505.41% (Supplementary Table S6, Supplementary Figure S6).

### 3.8. Forecasts of PD-Related Mortality (Figure 4 Shows Overall Trend of AAMR and Forecasted AAMR till 2030)

The optimal ARIMA models were chosen based on the lowest BIC and a satisfactory Ljung–Box test, indicating that the residuals are independently distributed. This was preferred as it minimized information loss while adequately capturing the underlying trend in the data. For our study, the ARIMA model (0, 1, 0), with a BIC of 38.39, was selected, as it provided a good fit to the time series data of age-adjusted rates. The Ljung–Box test indicated that the residuals were independently distributed (*p* = 0.3117). The model was cross-validated using a time-series cross-validation approach, and the average Root Mean Squared Error (RMSE) was 8.31. The model was then used to forecast age-adjusted rates from 2020 to 2030. The forecasted rate for 2020 was 100.26 (95% CI: 96.93–103.58), with a projected increase to 115.81 (95% CI: 104.78–126.84) by 2030. The forecast shows a consistent upward trend in the rates (Figure 4; Supplementary Table S7).

**Figure 3:**
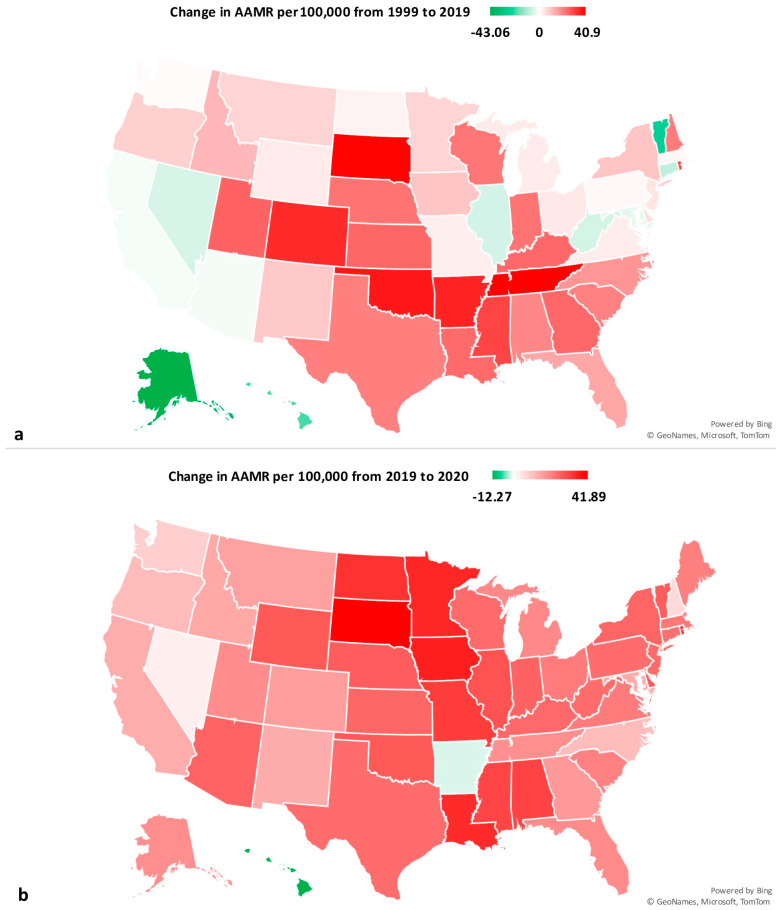
State level change in Parkinson disease mortality in the US (**a**) from 1999 to 2019; (**b**) from 2019 to 2020.

**Figure 4:**
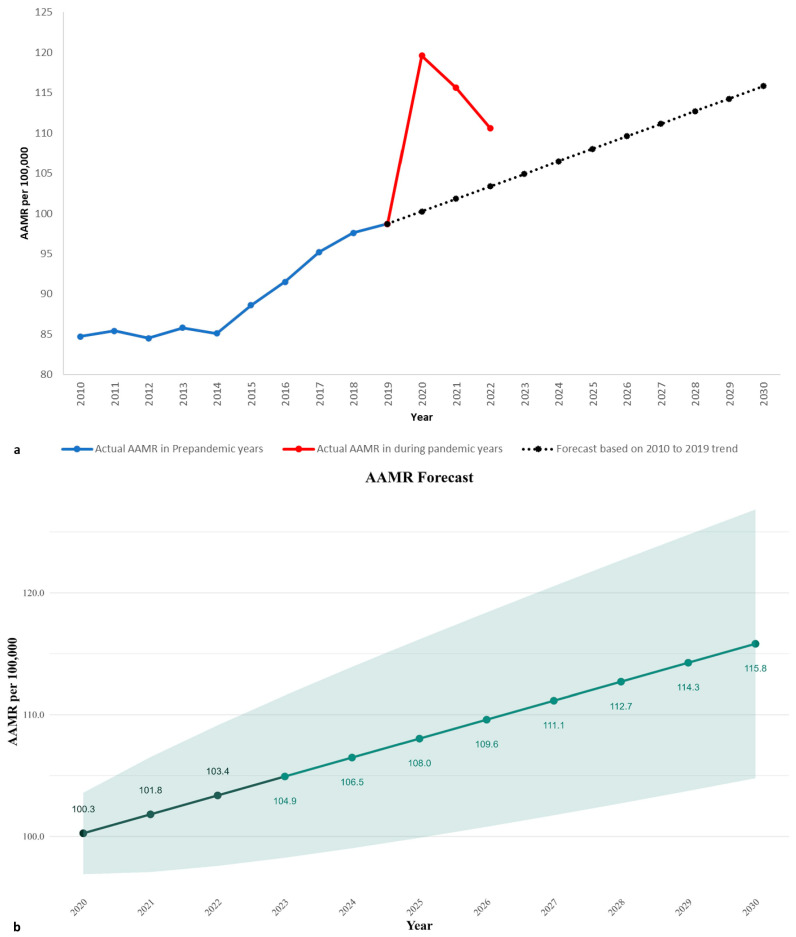
(**a**) Overall Parkinson’s disease AAMR from 1999 to 2022 when COVID-19 pandemic years spike highlighted in red line and forecasted AAMR based on pre-pandemic trend till 2030. (**b**) shows forecasted AAMR with 95% confidence intervals in green highlighted area.

## 4. Discussion

### 4.1. Overall

The total number of annual PD-related deaths doubled from the year 1999 to 2020. As the second most common neurodegenerative disease, PD and its epidemiology are immensely important. Understanding disease burdens in the US alerts health professionals and policymakers to areas for healthcare improvement. 

One theory behind the overall rise in PD mortality is that the disease burden grows as populations live longer. PD is a disease that presents with advanced age. Most patients are diagnosed between the ages of 55 and 65, with 9.34 cases per 1000 individuals older than 60 [7]. As Americans live longer, PD will become more commonplace, with some experts predicting its prevalence to double within the next two decades [21]. Additionally, the growing awareness of PD may also play a role in its increased incidence and mortality, as people are more inclined to visit the doctor when faced with new or unfamiliar symptoms.

Additionally, the overall mortality trend saw a marked increase in the year 2020. PD, like many other chronic and acute conditions, was affected by delays in treatment due to COVID-19 likely contributing to the increased mortality we noticed in our study. Our results echo the conclusions published by other publications focused on the burden COVID-19 placed on those with PD and its sequential rise in mortality rates [22].

### 4.2. Sex-Related Disparities

Over the course of our study, male AAMR was more than double that of women every year. There are multiple factors that may account for these sex-related disparities. First, men are 1.5 times more likely to develop PD compared to women, mainly owing to differences in the nigrostriatal dopaminergic system, which is affected by hormonal, genetic and environmental factors [23]. For example, estrogens have been shown to be neuroprotective, delaying the onset of PD in women [23]. Second, men with PD have shown more severe motor symptoms with fast progression of the disease compared to women [24]. Finally, studies have also shown a more pronounced neuroinflammatory and immune response in men with PD compared to women, potentially contributing to the observed differences in mortality [25]. These aforementioned factors affect men and women differently, which could be the key to understanding this wide mortality margin between genders^.^

### 4.3. Rece/Ethnicity-Related Disparities

Regarding race, this study found NH White individuals had the highest mortality rates from PD. NH Black or African American individuals saw the lowest mortality among the ethnic/racial groups, with roughly half the AAMR of NH White individuals each year. Multiple interpretations may account for these findings, such as those related to healthcare access, and socioeconomic and environmental factors. Individuals with lower socioeconomic status have limited access to specialized neurological care and advanced treatment options, including deep brain stimulation [26,27]. Additionally, this limited access to specialized neurologic care can lead to delayed diagnosis or under-reporting of PD in Black individuals [26]. Consequently, the lower observed mortality rates among Black individuals may not accurately reflect the true burden of PD in this population. Instead, these disparities might indicate an underestimation of PD-related mortality, driven by inadequate diagnosis and reporting practices. These data can be valuable for policymakers to target and mitigate these disparities.

### 4.4. Region-Based Disparities

Across census regions, there was an increase in PD AAMR across every region from 1999 to 2022. The greatest increase was observed in the South census region, but the overall greatest rates in AAMR were found in the Midwest. This may be explained by the reported relationship between pesticide exposure and PD [28]. The Midwest predominantly contributes to agriculture, with the greatest number of farm operations and greatest number of acres dedicated to farming compared to any other region [29]. However, this trend in AAMR being the highest in the Midwest may also be explained by racial differences across census regions. It is well established in this study and previous literature that PD death rates are highest among NH White individuals. Across the U.S., the NH White population in 2020 was 57.8%, while the percentage in the Midwest was 72.6% [30]. This greater proportion of NH White individuals may be related to greater AAMR in the Midwest region. 

### 4.5. State-Level Differences

Our study demonstrates how state trends have changed over time. In 2020, the five states with the largest AAMRs included Nebraska, Minnesota, South Dakota, Oklahoma and Iowa. Evaluating these trends may shed light on gaps in care in certain regions. Nebraska has consistently been one of the states with the highest AAMR over time [31]. This may be related to the rural makeup of Nebraska. Indeed, studies showed that being involved with farming activities and living in rural region carries a higher risk for developing PD [32]. Today, in 2024, ongoing research into the relationship between pesticides and PD is being conducted that may further explain the relationship between higher prevalence of PD in rural agricultural regions. A preliminary study conducted by the Barrow Neurological institute identified 14 pesticides associated with PD across the Great Plains, including the states Nebraska, Utah, Kansas, Colorado, Idaho, New Mexico, North Dakota, Oklahoma, South Dakota, Texas, Utah and Wyoming [28]. While this research is still in its early stages, it may contribute to understanding why certain states remain amongst the highest for AAMR for PD.

### 4.6. Location of PD-Related Death

Trends in location of PD deaths were examined from 1999 to 2022, with most deaths occurring outside medical facilities. From 1999 to 2022, there was a large increase in deaths at home. In 2020 specifically, there was a spike in overall AAMR, along with an increase in deaths at home, with 15,901 in 2019 compared to 21,277 in 2020. This trend may be explained, in part, by the COVID-19 pandemic, which saw a significant increase in home deaths across countries and age groups [33]. Throughout the pandemic, there was an increase in all-cause mortality across the world, which placed great strain on hospitals and decreased their ability to accommodate patients. Furthermore, families had to decide to weigh the risks of seeking hospital care for worsening chronic conditions, like PD, because of the threat of infection with COVID-19 at health facilities [33]. Beyond the influence of the COVID-19 pandemic, many people prefer to die at home rather than the hospital, and this may explain the trends in a larger proportion of home deaths even prior to the pandemic [34]. Finally, because of the chronic nature of PD, families may have more time for advanced care planning, deciding to facilitate end of life care at home, further increasing the rate of at-home deaths compared to deaths in healthcare facilities [35].

### 4.7. Forecasts of PD-Related Mortality

Forecasts of mortality rates using machine learning have proven to be of important utility in public health planning and for policymakers [36]. Our findings on forecasts of PD-related mortality rates show a steady increase in the AAMR. This is a concerning finding and may pose a serious healthcare challenge in the future.

### 4.8. Future Directions and Public Health Implications

The findings of this study highlight the growing burden of PD in the United States. This aligns with other studies examining the global burden of PD [37,38]. Data on disparities in PD-related mortality and future projections can assist healthcare policymakers in formulating plans to tackle these challenges. Forecast data from this study are consistent with studies suggesting that the aging population and increased survival of patients with PD may lead to an increased burden of PD in the future [39,40].

### 4.9. Limitations

This study still has limitations. The data gathered from CDC-WONDER are based on death certificate data. There can be various reporting biases or differences in cause-of-death attributions across institutions and individuals. The data provided in this study do not provide in-depth information on individuals with PD, duration of disease, or treatment received. Additionally, given that this database is based on ICD codes, it can be subject to misclassification bias, and we were unable to control for confounding factors.

## 5. Conclusions

This study makes important contributions to understanding trends in PD-related deaths over time across age, gender, race, states, and regions. Future investigation could incorporate more descriptive clinical data on individuals to further analyze trends in mortality across treatment, location, and risk exposures. Additionally, this study focused on the age group including individuals 65 and older. To continue to monitor trends in PD related deaths over time, future studies may investigate PD mortality among younger age groups. Overall, this study utilizes CDC WONDER, which presents reliable reporting on mortality data across the US, allowing this investigation to better describe trends in PD mortality over time while shedding light on areas of further investigation. Our findings highlight the increasing rate of PD AAMR and how it may become even more prevalent with time, emphasizing the value in increasing knowledge surrounding the disease and its trends to better prepare health systems and individual families for the burden of PD.

## Data Availability

The data compiled in this manuscript are publicly available upon request from the CDC WONDER database.

## Supplementary Materials

The following supporting information can be downloaded at: https://www.mdpi.com/article/10.3390/neurosci6010006/s1, Table S1. Parkinson’s Disease age-adjusted mortality rate per 100,000 people; overall and stratified by gender, 1999–2022; Table S2. Parkinson’s Disease age-adjusted mortality rate per 100,000 people; stratified by race, 1999–2022. Table S3. Parkinson’s Disease age adjusted mortality rate per 100,000 people; stratified by census region, 1999–2022. Table S4. Parkinson’s Disease crude mortality rate per 100,000 people; stratified by age group, 1999–2022. Table S5. Parkinson’s Disease age adjusted mortality rate per 100,000 people; stratified by state, 1999–2022. Table S6. Parkinson’s Disease age adjusted mortality rate per 100,000 people; stratified by place of death, 1999–2022. Table S7. Forecasts of Parkinson Disease-related Age-adjusted mortality rate using machine learning (AAMR), 2010–2030. Figure S1. Joinpoint model of Parkinson’s Disease related AAMR per 100,000 people overall and stratified by gender, 1999–2022. Figure S2. Joinpoint model of Parkinson’s Disease related AAMR per 100,000 people stratified by race, 1999–2022 Figure S3. Joinpoint model of Parkinson’s Disease related AAMR per 100,000 people overall and stratified by census region, 1999–2022. Figure S4. State-level change in Parkinson’s disease AAMR from 1999–2019. Figure S5. State-level change in Parkinson’s disease AAMR from 2019 to 2020. Figure S6. Parkinson’s Disease annual deaths; stratified by place of death, 1999–2022.

## Glossary

Abbreviations: Parkinson’s Disease (PD), CDC Wide-ranging Online Data for Epidemiologic Research (CDC WONDER), Age-Adjusted Mortality Rate (AAMR), International Classification of Diseases (ICD), Non-Hispanic (NH), Annual Percent Change (APC), Confidence Interval (CI), Average Annual Percent Change (AAPC).
