# Supplementary material for: Trends and Disparities in Parkinson’s Disease Mortality in the United States with Predictions Using Machine Learning
Source: NeuroSci. 2025 Jan 15;6(1):6. doi: 10.3390/neurosci6010006 (PMC11755521; doi:10.3390/neurosci6010006)
Supplement: Supplementary file 1 [file neurosci-06-00006-s001.zip › neurosci-3408235-supplementary.pdf]

NeuroSci

MDPI

Supplementary

Supplemental Table S1. Parkinson's Disease age-adjusted mortality rate per 100,000 people; overall and stratified by gender, 1999-2022.

Year	Overall	Female	Male	Difference
1999	88.9	61	138.2	77.2
2000	91.3	62.9	142.2	79.3
2001	91.3	62.7	142	79.3
2002	92	63.5	142.2	78.7
2003	92.8	64.1	142.4	78.3
2004	90	61.5	138.4	76.9
2005	93.8	64.5	143.3	78.8
2006	88.3	60	135.2	75.2
2007	87.2	59.6	132.5	72.9
2008	85.4	57.8	130.3	72.5
2009	82.5	55.2	126.6	71.4
2010	84.7	57.1	128.6	71.5
2011	85.4	57.3	129.4	72.1
2012	84.5	56.1	128.4	72.3
2013	85.8	56.1	131.1	75.0
2014	85.1	56.1	128.6	72.5
2015	88.6	58	133.9	75.9
2016	91.5	60.2	137.5	77.3
2017	95.2	62.3	143	80.7
2018	97.6	64.2	145.7	81.5
2019	98.7	64	148.3	84.3
2020	119.6	78.7	177.6	98.9
2021	115.6	75.9	171.4	95.5
2022	110.6	71.2	167.4	96.2
**Joinpoints (Year)**	1 (2014)	1 (2014)	1 (2014)	-
**APC-Segment 1 (95% CI)**	-0.61* (-1.17 to -0.17)	-0.95* (-1.61 to -0.45)	-0.81* (-1.36 to -0.32)	-
**APC-Segment 2 (95% CI)**	4.26* (3.30 to 5.55)	4.15* (2.99 to 5.88)	4.18* (3.23 to 5.54)	-
**Average APC (95% CI)**	1.06* (0.79 to 1.34)	0.79* (0.50 to 1.11)	0.90* (0.64 to 1.18)	-

Supplemental Table S2. Parkinson's Disease age-adjusted mortality rate per 100,000 people; stratified by race, 1999-2022.

Year	NH White	NH Black or African American	NH American Indian or Alaska Native	Hispanic or Latino
1999	94.5	44.84	64.8	60.63
2000	97.49	45.51	56.94	61.22
2001	97.37	44.67	62.5	66.5
2002	98.51	46.33	74.01	60.37
2003	99.14	48.01	77.48	62.68
2004	96.3	47.77	60.52	61.19
2005	100.5	48.61	68.29	69.58
2006	94.62	44.38	70.89	66.95
2007	93.55	46.69	65.83	62.46
2008	91.94	43.9	62.24	64.38
2009	89.11	41.12	59.21	60.01
2010	91.5	42.71	59.2	65.94
2011	92.43	43.24	61.65	62.64
2012	91.71	43.39	62.11	62.93
2013	92.86	46.09	55.27	67.23
2014	92.56	45.88	60.88	63.92
2015	96.24	47.37	61.96	68.37
2016	99.75	50.68	60.54	68.99
2017	103.92	53.15	71.52	71.08
2018	106.44	56.59	72.18	73.36
2019	108.2	54.8	67.64	73.37
2020	130.87	70.68	73.53	93.03
2021	128.09	65.12	73.05	85.23
2022	122.52	62.07	65.15	83.08
**Joinpoints (Year)**	1 (2014)	1 (2012)	1 (2013)	2 (2017, 2020)
**APC Segment 1 (95% CI)**	-0.48* (-1.02 to -0.04)	-0.71 (-2.08 to 0.25)	-0.86 (-7.51 to 0.72)	0.50 (-0.30 to 1.51)
**APC Segment 2 (95% CI)**	4.52* (3.56 to 5.86)	4.58* (3.47 to 6.25)	2.03* (0.24 to 8.94)	9.46 (-0.49 to 11.98)
**APC Segment 3 (95% CI)**	-	-	-	-3.15 (-8.24 to 4.58)
**Average APC (95% CI)**	1.23* (0.97 to 1.51)	1.56* (1.12 to 2.02)	0.26 (-0.51 to 1.12)	1.30* (0.87 to 1.85)

Supplemental Table S3. Parkinson's Disease age adjusted mortality rate per 100,000 people; stratified by census region, 1999-2022.

Year	Northeast	Midwest	South	West
1999	81.86	98.95	79.91	99.72
2000	85.58	99.97	84.15	99.45
2001	86.32	99.38	83.35	100.8
2002	85.34	102.54	84.4	99.83
2003	83.14	101.06	87.05	103
2004	81.78	99.05	83.55	98.79
2005	85.96	104.66	87.6	100.22
2006	79.6	96.51	82.59	97.27
2007	80.39	94.24	83.31	92.67
2008	77.35	94.63	79.7	92.77
2009	75.38	91.14	77.54	88.23
2010	77.61	91.58	81.15	90.35
2011	79.78	92.36	80.17	92.17
2012	75.56	93.11	80.33	90.61
2013	80.47	94.44	81.22	89.4
2014	78.18	94.47	80.98	88.38
2015	80.83	94.28	85.88	94.2
2016	82.61	98.3	89.36	95.72
2017	84.1	101.25	95.25	98.13
2018	87.72	105.03	96.98	99.52
2019	86.21	106.61	98.57	101.66
2020	110.09	133.08	119.02	115.36
2021	98.6	122.67	118.51	118.41
2022	93.37	118.14	113.4	113.11
**Joinpoints (Year)**	2 (2016, 2020)	2 (2016, 2020)	1 (2014)	1 (2014)
**APC Segment 1 (95% CI)**	-0.50 (-1.14 to 0.04)	-0.52* (-1.02 to -0.06)	-0.28 (-0.88 to 0.22)	-0.97* (-1.51 to -0.53)
**APC Segment 2 (95% CI)**	7.41 (-0.46 to 11.79)	8.00* (0.01 to 12.17)	5.16* (4.16 to 6.54)	3.64* (2.74 to 5.02)
**APC Segment 3 (95% CI)**	-4.76 (-10.40 to 3.17)	-2.93 (-7.58 to 4.08)	-	-
**Average APC (95% CI)**	0.45 (0.00 to 0.86)	0.70* (0.33 to 1.06)	1.58* (1.30 to 1.87)	0.61* (0.37 to 0.86)

Supplemental Table S4. Parkinson's Disease crude mortality rate per 100,000 people; stratified by age group, 1999-2022.

Year	65-74 Crude Rate	75-84 Crude Rate	85+ Crude Rate
1999	22.26	122.25	275.61
2000	22.28	125.95	284.91
2001	22.44	126.77	282.18
2002	23.12	124.43	292.07
2003	23.17	126.62	291.46
2004	22.07	123.8	281.27
2005	22.31	127.64	300.92
2006	21.02	120.75	280.91
2007	20.22	121.48	273.46
2008	20.42	117.63	269.16
2009	18.48	114.89	261.57
2010	19.23	117.82	268.32
2011	20.08	118.19	268.97
2012	19.2	117.14	268.44
2013	19.9	118.36	272.53
2014	19.9	117.37	269.56
2015	21.06	121.59	281
2016	21.99	124.56	291.65
2017	23.78	129.07	300.77
2018	24.71	132.05	308.41
2019	25	134.48	309.36
2020	31.46	163.64	367.58
2021	29.76	155.7	364.69
2022	30.09	152.36	332.6

Supplemental Table S5. Parkinson's Disease age adjusted mortality rate per 100,000 people; stratified by state, 1999-2022.

State	1999	2019	2020
Alabama	66.73	86.02	116.54
Alaska	113.07	70.01	88.69
Arizona	88.38	87.05	112.55
Arkansas	72.4	106.76	105.38
California	101.55	100.06	113.71
Colorado	90.74	124.21	140.21
Connecticut	88.43	80.16	103.95
Delaware	77.07	82.51	109.65
District of Columbia	66.64	78.63	93.17
Florida	67.92	82.34	101.51
Georgia	72.52	96.64	113.28
Hawaii	105.71	84.99	72.72
Idaho	105.57	117.25	131.85
Illinois	99.16	93.31	121.47
Indiana	93.21	115.52	141.17
Iowa	100.69	110.25	146.28
Kansas	94.92	119.35	144.26
Kentucky	86.72	110.89	135.52
Louisiana	69.06	92.88	126.99
Maine	102.6	102.64	123.72
Maryland	109.6	106.13	120.14
Massachusetts	87.24	88.58	110.78
Michigan	97.57	101.04	120.71
Minnesota	116.14	123.06	157.96
Mississippi	78.75	108.42	138.5
Missouri	93.54	96.49	128.05
Montana	94.07	101.01	116.64
Nebraska	114.32	136.62	163.08
Nevada	85.28	79.59	82.28
New Hampshire	91.2	112.32	118.53
New Jersey	80.58	84.9	109.1
New Mexico	85.69	94.52	108.35
New York	65.83	75.36	100.63
North Carolina	80.35	97.37	108.64

North Dakota	104.39	106.32	139.57
Ohio	101.74	105.54	127.24
Oklahoma	83.36	119.59	146.54
Oregon	104.96	112.59	123.85
Pennsylvania	94.76	95.82	119.66
Rhode Island	72.7	102.55	134.69
South Carolina	76.93	97.19	118.01
South Dakota	73.76	113.59	155.48
Tennessee	77.3	118.2	136.45
Texas	92.68	113.36	137.43
Utah	101.01	126.17	144.99
Vermont	134.49	108.54	135.22
Virginia	89.91	93.02	114.67
Washington	110.65	111.19	119.38
West Virginia	96.81	90.89	114.92
Wisconsin	90.41	112.62	137.09
Wyoming	79.24	82.48	109.44

Supplemental Table S6. Parkinson's Disease age adjusted mortality rate per 100,000 people; stratified by place of death, 1999-2022.

Year	Medical Facility - Inpatient	Medical Facility - Outpatient or ER	Medical Facility - Dead on Arrival	Decedent's home	Hospice facility	Nursing home/long term care	Other
1999	7714	946	228	4936	-	15740	836
2000	7804	987	226	5417	-	16147	1061
2001	7847	961	176	5601	-	16332	1235
2002	7668	954	170	5878	-	16713	1400
2003	7522	920	138	6408	74	16882	1648
2004	7074	879	110	6804	147	16198	1712
2005	7385	901	97	7212	576	17211	1570
2006	6957	876	94	7226	714	16033	1460
2007	6775	821	82	7382	1111	15848	1535
2008	6463	753	62	7470	1234	15555	1613
2009	5702	854	47	7899	1259	14787	1761
2010	5784	856	52	8701	1741	15240	1919
2011	5876	858	57	9170	1966	15519	2052
2012	5432	854	59	9831	2228	15278	2255

2013	5405	868	67	10558	2506	15481	2498
2014	5258	893	41	11009	2796	15702	2210
2015	5407	848	53	11936	3296	16552	2296
2016	5506	890	43	13144	3514	17016	2519
2017	5650	942	47	13923	3828	18353	2743
2018	5971	968	52	15203	4055	18807	2982
2019	6094	968	37	15901	4218	19406	3156
2020	7829	1164	45	21277	4438	22833	4045
2021	7562	1075	41	21992	4618	17893	3858
2022	7774	1058	38	21425	4814	18951	4373

Supplemental Table S7. Forecasts of Parkinson Disease-related Age-adjusted mortality rate using machine learning (AAMR), 2010-2030. .

Year	Actual AAMR in Prepandemic years	Actual AAMR in during pandemic years	Forecast based on 2010 to 2019 trend
**2010**	**84.7**		
2011	85.4		
2012	84.5		
2013	85.8		
2014	85.1		
2015	88.6		
2016	91.5		
2017	95.2		
2018	97.6		
2019	98.7	98.7	98.7
2020		119.6	100.2556
2021		115.6	101.8111
2022		110.6	103.3667
2023			104.9222
2024			106.4778
2025			108.0333
2026			109.5889
2027			111.1444
2028			112.7
2029			114.2556
2030			115.8111

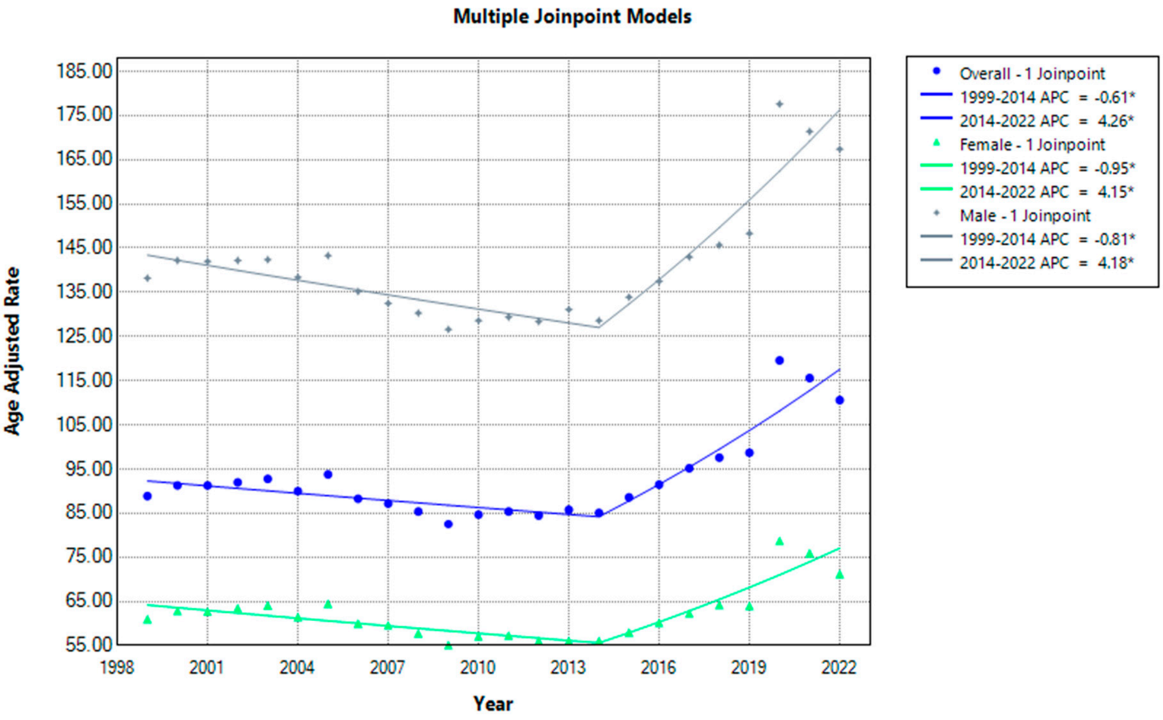

Supplemental Figure S1. Joinpoint model of Parkinson's Disease related AAMR per 100,000 people overall and stratified by gender, 1999-2022 (* indicates the APC is statistically significant).

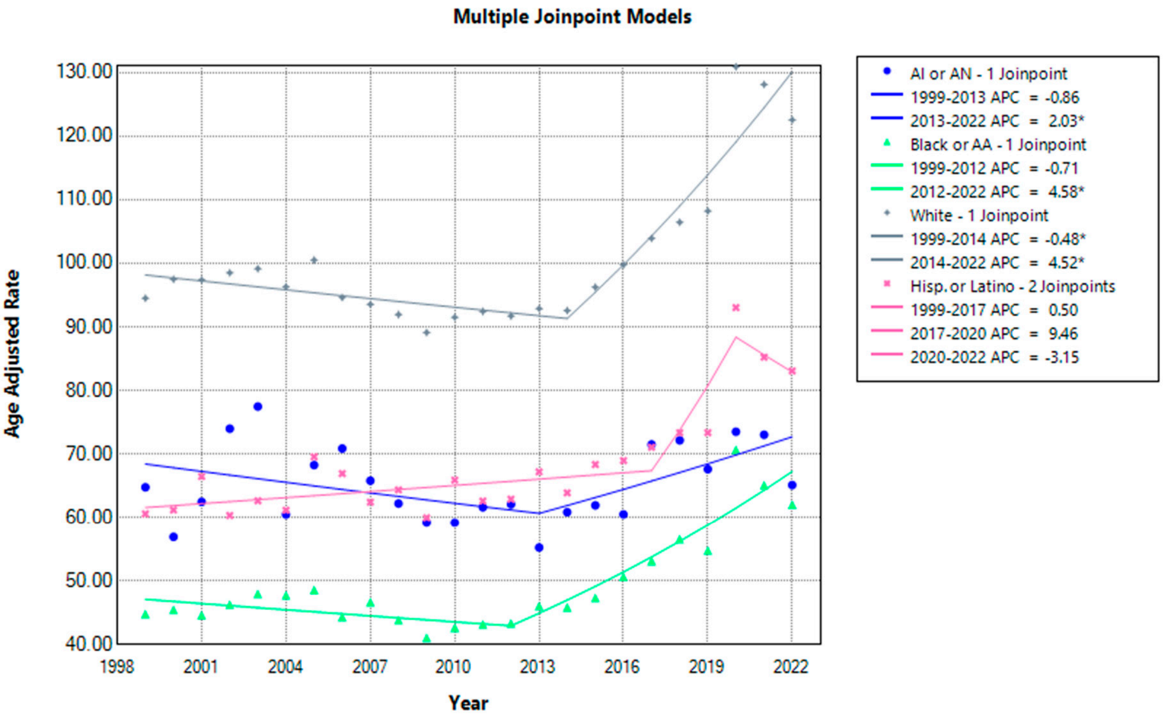

Supplemental Figure S2. Joinpoint model of Parkinson's Disease related AAMR per 100,000 people stratified by race, 1999-2022 (* indicates the APC is statistically significant).

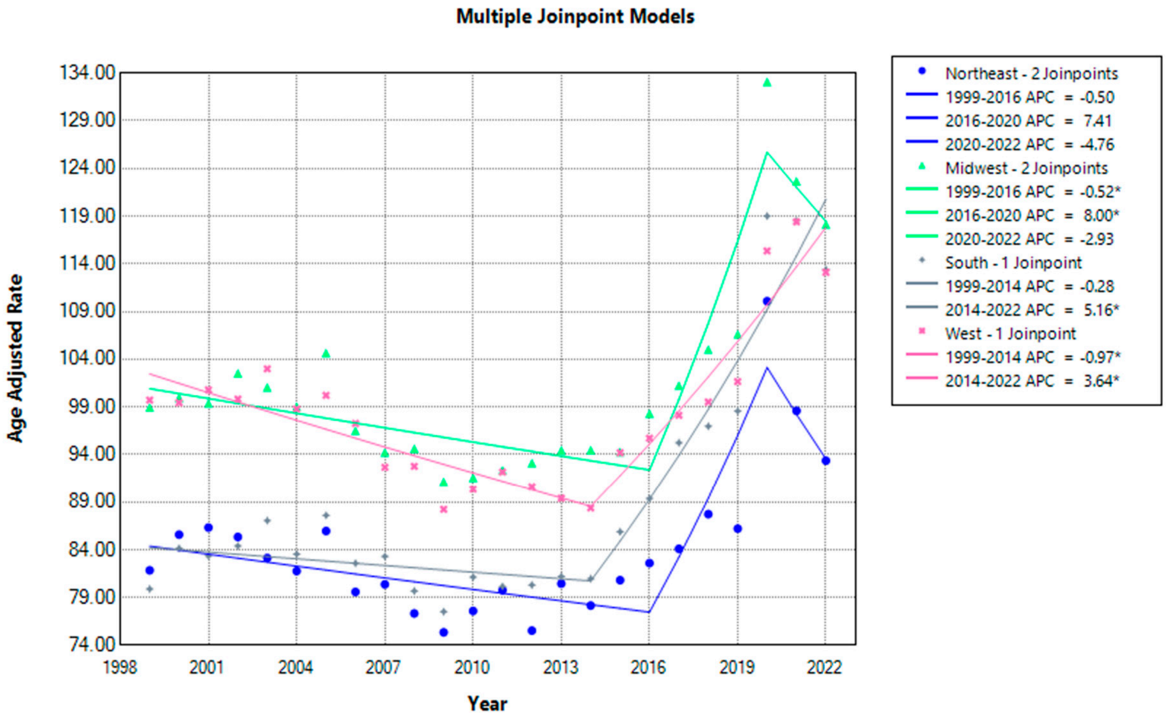

Supplemental Figure S3. Joinpoint model of Parkinson's Disease related AAMR per 100,000 people overall and stratified by census region, 1999-2022 (* indicates the APC is statistically significant).

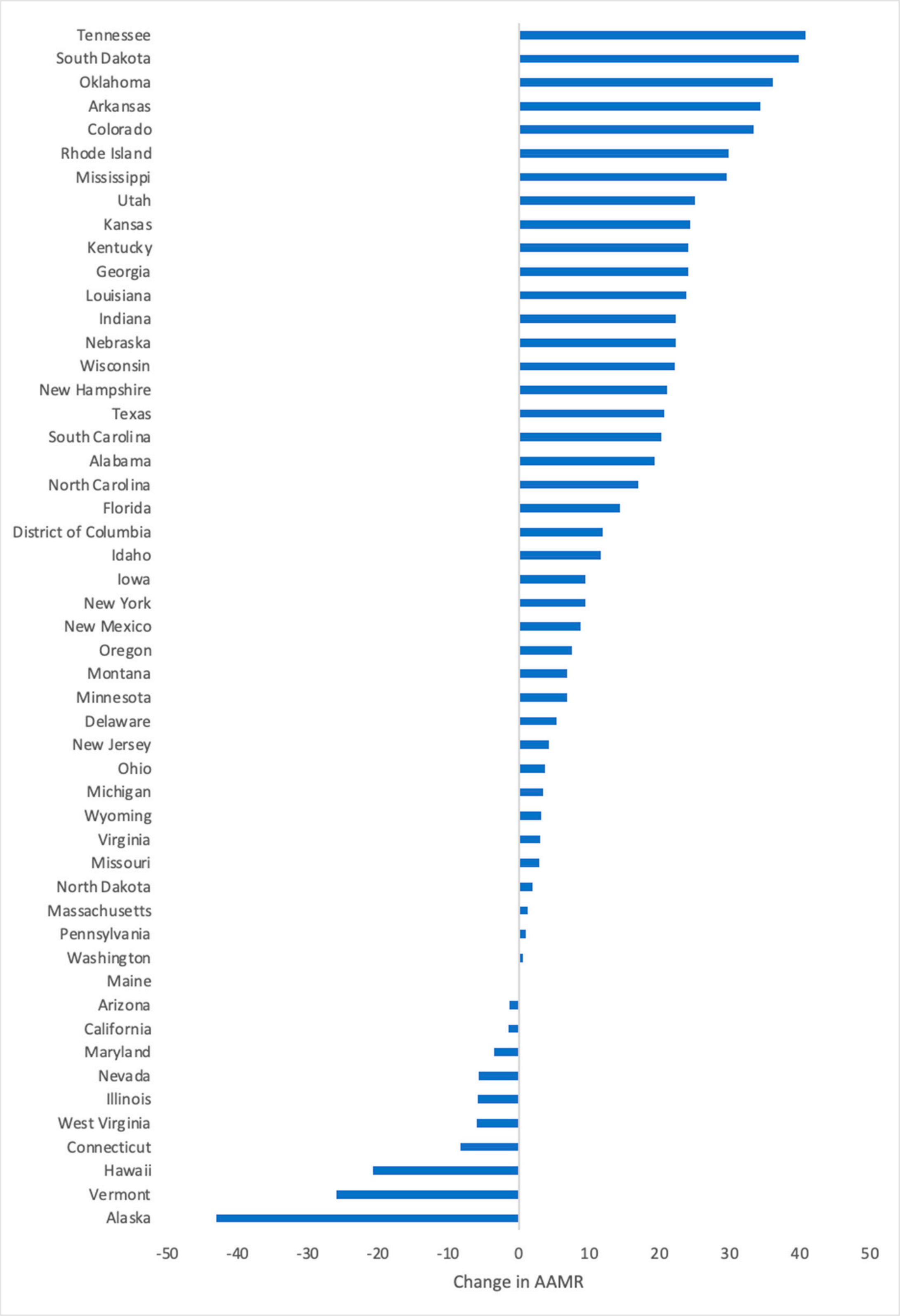

Supplemental Figure S4. State-level change in Parkinson's disease AAMR from 1999-2019.

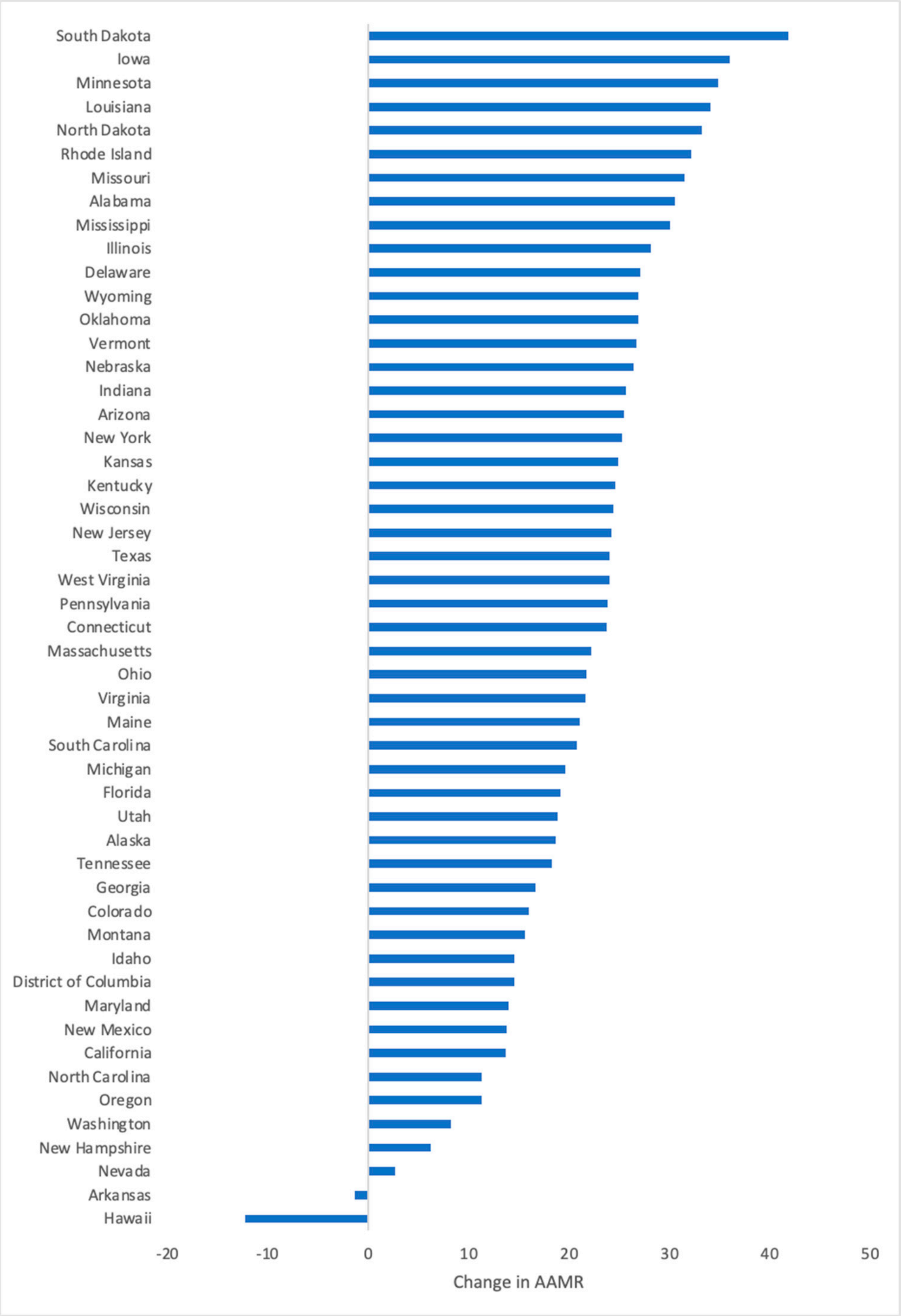

Supplemental Figure S5. State-level change in Parkinson's disease AAMR from 2019 to 2020.

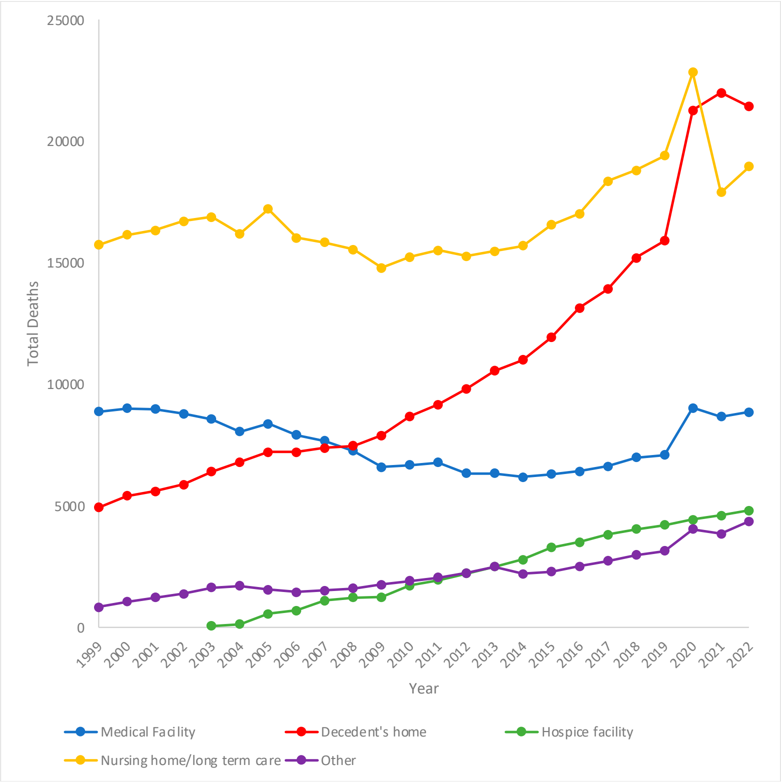

Supplemental Figure S6. Parkinson's Disease annual deaths; stratified by place of death, 1999-2022.
